# Discs Large Homolog 1 Splice Variants Regulate p38 –Dependent and –Independent Effector Functions in CD8+ T Cells

**DOI:** 10.1371/journal.pone.0133353

**Published:** 2015-07-17

**Authors:** Oscar Silva, Jillian Crocetti, Lisa A. Humphries, Janis K. Burkhardt, M. Carrie Miceli

**Affiliations:** 1 Department of Microbiology, Immunology and Molecular Genetics, University of California Los Angeles, Los Angeles, California, United States of America; 2 Molecular Biology Interdepartmental Program, University of California Los Angeles, Los Angeles, California, United States of America; 3 Department of Laboratory Medicine, Children’s Hospital of Philadelphia and University of Pennsylvania, Philadelphia, Pennsylvania, United States of America; Oklahoma Medical Research Foundation, UNITED STATES

## Abstract

Functionally diverse CD8+ T cells develop in response to antigenic stimulation with differing capacities to couple TCR engagement to downstream signals and functions. However, mechanisms of diversifying TCR signaling are largely uncharacterized. Here we identified two alternative splice variants of scaffold protein Dlg1, Dlg1AB and Dlg1B, that diversify signaling to regulate p38 –dependent and –independent effector functions in CD8+ T cells. Dlg1AB, but not Dlg1B associated with Lck, coupling TCR stimulation to p38 activation and proinflammatory cytokine production. Conversely, both Dlg1AB and Dlg1B mediated p38-independent degranulation. Degranulation depended on a Dlg1 fragment containing an intact Dlg1SH3-domain and required the SH3-ligand WASp. Further, Dlg1 controlled WASp activation by promoting TCR-triggered conformational opening of WASp. Collectively, our data support a model where Dlg1 regulates p38-dependent proinflammatory cytokine production and p38-independent cytotoxic granule release through the utilization of alternative splice variants, providing a mechanism whereby TCR engagement couples downstream signals to unique effector functions in CD8+ T cells.

## Introduction

CD8+ cytotoxic T lymphocytes (CTLs) are critical components of the adaptive immune response due to their ability to produce proinflammatory cytokines and induce target cell killing through lytic factor degranulation. Although these distinct CTL functions are often required to efficiently clear intracellular pathogens, they are not always coordinately invoked [1]. In fact, CD8+ CTLs can selectively degranulate but not produce proinflammatory cytokines, or can concurrently degranulate and produce proinflammatory cytokines depending on the concentration of antigen or the type of antigen presenting cell present at a localized tissue microenvironment [1, 2]. Furthermore, during an adaptive immune response functionally diverse CD8+ CTLs develop with differential capacities to express a spectrum of cytokines and lytic factors in order to selectively orchestrate inflammation and target cell killing [3]. Such functional diversity, and selectivity suggest that signaling complexes downstream of the T cell receptor (TCR) may be differentially employed to diversify CD8+ T cell functionality. However, mechanisms by which TCR engagement is linked to select downstream signals and functions remains poorly understood.

Scaffold proteins have emerged as key molecular intermediates coupling extracellular receptors to intracellular signaling pathways, and thus are key conduits for specifying TCR signaling and functional outcome [4]. Discs large homolog 1 (Dlg1), a membrane associated guanylate kinase (MAGUK) scaffold protein co-localizes with the TCR complex at the immunological synapse (IS) during T cell activation [5, 6]. Dlg1 coordinates the TCR-induced alternative p38 pathway by juxtaposing tyrosine kinases Lck and ZAP70 with p38 mitogen-activated protein kinase (MAPK) [7, 8]. In this molecular complex, Dlg1 bridges Lck and ZAP70, allowing for Lck-dependent ZAP70 activation and ultimately direct ZAP70 phosphorylation of p38 [8, 9]. This pathway leads to select activation of NFAT, but not NFκB, through S54 phosphorylation of NFATc2; thereby coupling proximal TCR proximal kinases (Lck and ZAP70), to a subset of potential TCR signaling outputs [8]. Additionally, Dlg1 controls antigen-induced F-actin polymerization, polarized TCR and lipid raft synaptic clustering, MTOC orientation and cytotoxicity in CD8+ CTLs [5, 10]. Recently, Dlg1 has been shown to regulate the development of antigen-experienced T cells, Treg, Thelper and memory T cell subsets [11–14]. In human CD4+ Tregs, Dlg1 also controls PTEN stabilization and Akt activation [13]. However, precisely how Dlg1 couples to downstream TCR signaling pathways and cytoskeletal dynamics and how these activities impact T cell functionality has yet to be elucidated.

Structurally, Dlg1 contains: three PSD95/Dlg/ZO-1 (PDZ) domains, a Src homology 3 (SH3) domain and a guanylate kinase (GUK) domain. In addition, Dlg1 has four known areas of alternative splicing: a site in the 5′UTR that regulates *dlg1* translation; a proline-rich region upstream of PDZ1 that can contain the i1A and/or i1B domains; a region between SH3 and GUK, known as the HOOK domain; that can contain exons i3, i2, i5 and/or i4; and an N-terminal region that can contain either a CXCα palmitoylation domain or L27β oligomerization domain [15–18]. The exons encoded within the HOOK domain are numerically non-sequential as they were named based on when they were identified, rather than their actual germline genomic order. Characterization of Dlg1 splice variants in epithelial, neuronal and cardiac cells demonstrates that a subset of possible variants are expressed in each cell type, However, which Dlg1 splice variants are expressed in T cells, and the role that these variants play in coordinating T cell signaling has yet to be examined. [16, 18, 19].

Here we report that CD8+ T cells utilize Dlg1 splice variants to couple TCR engagement to proinflammatory cytokine production and/or degranulation. We found two major Dlg1 splice variants to be expressed in T cells: Dlg1 L27β-i1Ai1B-i3i5 (Dlg1AB) and Dlg1-L27β-i1B-i3i5 (Dlg1B). Dlg1AB, but not Dlg1B induced proinflammatory cytokine production by associating with Lck and promoting alternative p38 activation and NFAT-dependent gene expression of IFNγ and TNFα. Conversely, both Dlg1AB and Dlg1B promoted p38-independent lytic factor degranulation, which depended on Dlg1 fragments containing an intact SH3-domain and required WASp. Dlg1 controlled WASp activation by promoting TCR-triggered opening of WASp. Together, our findings demonstrate that Dlg1 plays a critical role in proinflammatory cytokine production and cytotoxicity, and identifies the expression of Dlg1 splice variants as one possible mechanism to diversify T cell functionality by regulating the formation of p38-dependent and p38-independent signaling complexes.

## Materials and Methods

### Ethics Statement

This study was carried out in strict accordance with the recommendations in the Guide for the Care and Use of Laboratory Animals of the National Institute of Health. The protocol was approved by the Office of Animal Research Oversight and the Chancellor’s Animal Research Committee (Permit Number: ARC 1996–155). The Chancellor’s Animal Research Committee serves as UCLA’s Institutional Animal Care and Use Committee (IACUC) and upholds the legal and ethical standards of animal care at UCLA. All mice used were monitored daily and euthanized by inhalation of carbon dioxide at 8–14 weeks of age.

### DNA Constructs

Retroviral constructs pMSCV-GFP-Puro (MGP) and pMSCV-IRES-GFP (MIG) have been previously described [20, 21]. GFP was removed from the MGP construct and replaced with dsRED using the BglII and NotI restriction sites, this vector was named MRP. The Invitrogen Block-IT miR RNAi Designer was used to predict siRNA sequences against WASp (TGTGTGCTTCGTGAAGGATA or ACCCTCAGAAGTCCTACTTCA) or specific Dlg1 regions: L27β (TTCCATAGAGCGGGTTATTAA), i1A (CCAGTCCCTGCTGAGAGTACT), i1A* (TCCCTGCTGAGAGTACTGTCG), i1B (AGCTTAGAGACACCAACTTAT), i3 (AAGAACCTCTTTTCCCGAAAA), i3* (GAACCTCTTTTCCCGAAAATT), 3′UTR (GTCCTCCACACTGACACAGAT). These sequences were cloned into the MGP or MRP microRNA-based knockdown vectors using a previous published strategy [21]. To generate MIG-Dlg1 splice variant constructs PCR amplified Dlg1 L27β-i1Ai1B-i2i5 from [8] was cloned into MIG using XhoI and EcoRI restriction sites. Next, cDNA from primary CD8+ OT-1 CTLs was used to amplify N-terminal Dlg1 fragments containing the i1A/i1B splice region or C-terminal Dlg1 fragments containing the i2/i3/i4/i5 splice region and cloned into MIG-Dlg1 L27β-i1A-i1B i2i5 using either XhoI/BstBI (N-terminal fragments) or BstBI/EcoRI (C-terminal fragments) restriction sites. Once all four possible Dlg1 splice variant combinations were cloned into MIG, Dlg1 L27β-i1Ai1B-i3i5 (Dlg1 AB) and Dlg1-L27β-i1B-i3i5 (Dlg1 B) were cloned into pGEX 4T-1 using EcoRI and SalI restriction sites to generate GST-constructs. To generate MIG-Dlg1 i1B truncation constructs, Dlg1-L27β-i1B-i3i5 was used as a template and an XhoI forward primer with different EcoRI reverse primers that terminated the Dlg1 sequence at various lengths were used. The primer sets used to generate MIG-Dlg1 splice variant, MIG-Dlg1 truncation, and GST-Dlg1 constructs can be found in S1 Table.

### Antibodies and Reagents

For T cell stimulations anti-CD3, clone 145-2C11 (BD 553057) and anti-CD28, clone 37.51 (BD 553295) were used. For immunoprecipitations (IP) and immunoblotting (IB): Mouse anti-Dlg1 (BD 610875), Rabbit anti-p38, clone C20 (Santa Cruz Biotechnology sc-535), Mouse anti-Lck, clone 3A5 (Santa Cruz Biotechnology sc-433), Mouse anti-ZAP70 (BD 610239) Mouse anti-WASp, clone B-9 (Santa Cruz Biotechnology, sc-13139), Rabbit anti-phospho-p38 (T180/Y182), clone D3F9 (Cell Signaling 4511), Donkey Anti-Mouse IgG-HRP (Jackson ImmunoResearch 715-035-150), and Donkey-Anti-Rabbit IgG-HRP (Santa Cruz Biotechnology sc-2305) were used. For flow cytometry, Rat anti-CD8b-PE, clone H35-17.2 (BD 550798), Alexa Fluor647 Mouse anti-p38 MAPK (pT180/pY182), clone 36 (BD 612595), Rat anti-IFNγ-APC, clone XMG1.2 (BD 554413), Rat anti-IL-2-APC, clone JES6-5H4 (BD 554429), Rat anti-TNFα-APC (BD 554420), Rat anti-CD107a-APC, clone 1D4B (BD 560646), Alexa Fluor647 Phalloidin (Molecular Probes, Invitrogen A22287), Mouse monoclonal anti-WASP 26E6 [22] and Alexa Fluor647 AffiniPure F(ab)2 Donkey Anti-Mouse IgG (Jackson ImmunoResearch 715-606-150) were used. For inhibitors studies Insolution SB203580 (Calbiochem 559398), cytochalasin D (Sigma C8273) and DMSO (Sigma D2650) were used. For antigen experiments OVA_257-264_ peptide from AnaSpec (#60193) was used.

### Cell Culture

Spleen and lymph node cells were obtained from 8-to 14-week old OT-1 mice [23]. These mice have CD8+ T cells that recognize OVA_257-264_ in the context of H-2K^b^. CD8+ cells were sorted using CD8a Ly-2 microbeads (Miltenyi 130-049-401) and stimulated in vitro with plate-bound anti-CD3/anti-CD28 or MEF.B7.OVA antigen presenting cells for 48–72hrs in complete media composed of RPMI 1640 medium with 10% FCS, sodium pyruvate, 50nM β-mercaptoethanol, penicillin, streptomycin and glutamine followed by expansion in rIL-2 (100 units/mL) for an additional 3–4 days to generate primary mouse CTLs. BI-141 murine hybridoma cells were maintained in complete media. OT-1 hybridoma cells were maintained as in [8]. MEF.B7.OVA cells stably expressing H-2K^b^, B7.1, and OVA_257-264_ where maintained as in [5]. H-2K^b^ EG.7 cells constitutively expressing OVA_257-264_ and EL-4 cells were maintained in as in [24].

### Retrovirus Production and Infections

293T cells were transfected with pCL-Eco and knockdown or overexpression constructs using TransIT 293 (Mirus 2705) per manufacturer’s instructions. After 48hrs, viral supernatant was harvested, filtered and used to infect activated primary OT-1 cells or hybridomas via spin infection in the presence of polybrene (8μg/mL) at 1250*g* for 90mins at room temperature. For primary cell infections, T cells were infected after 48–72hrs of stimulation with anti-CD3/anti-CD28. Two spin-infections were performed on cells 24hrs apart. Functional assays were performed 24–48hrs after the last spin-infection.

### RT-PCR and qPCR

T cells were stimulated with platebound anti-CD3 (2μg/mL) and anti-CD28 (2μg/mL) antibodies or left unstimulated. RNA was isolated using TRIZOL reagent. RNA (2μg) was reverse-transcribed using Superscript III (Invitrogen) according to the manufacture’s instructions with oligo(dT) primer in 20μL reactions. cDNA was diluted 1:5 with DEPC-H20 and used for subsequent RT-PCR and qPCR analysis. For RT-PCR, 2.0 μL of cDNA was used for PCR amplification with annealing temperatures of 62°C and 58°C for the i1A/i1B and i2/i3 primer sets respectfully. For qPCR, Sybrgreen-based quantitative PCR analysis was performed on the iCycler BioRad instrument according to manufacturer’s instructions (BioRad). Amplification conditions were: 95°C for 3 minutes, followed by 40 cycles of 95°C for 30 seconds and 60°C for 30 seconds. For all experiments, mRNA was normalized to L32. Primers used for RT-PCR and qPCR analysis can be found in S1 Table.

### DNA Sequencing

PCR products from RT-PCRs were gel extracted using the QIAquick Gel Extraction kit by QIAGEN. Purified products were sequenced by Laragen Sequencing and Genotyping Service; Culver City, CA

### GST-pulldowns, Immunoprecipitations and Immunoblotting

Stimulated OT-1 hybridoma lysates for IPs and pulldowns were generated by incubating 4 x10^7^ cells with anti-CD3 and anti-CD28 followed by antibody crosslinking using donkey anti-hamster secondary for 15mins at 37°C. Cells were lysed in the presence of protease and phosphatase inhibitors and cleared by centrifugation. For GST pulldown assays, lysates from stimulated or unstimulated OT-1 hybridoma cells were incubated for 2hrs at 4°C with purified GST-alone or GST-Dlg1 fusion proteins and then washed. IPs were performed as published [8]. Proteins were separated by SDS-PAGE and transferred to nitrocellulose. Membranes, blocked with TBS plus 5% milk and 0.1% Tween-20, were incubated with primary antibodies (1:1000) overnight at 4°C, followed by incubation with HRP-conjugated secondary antibodies (1:5000) for either 4hrs (Dlg1 blots) or 1hr (all others) at room temperature. Signals were detected by chemiluminescence reagents (Pierce Western Lightening *Plus-*ECL). Quantification by densitometry was done using ImageJ.

### ELISA

To detect IFNγ, TNFα, IL-2, and Granzyme B protein in supernatants, ELISAs were performed with Ready-Set-Go kits from eBioscience (88–7334, 88–7324, 88–7024, 88–8022) according to the manufacturer’s instructions.

### Intracellular Dlg1 and WASp

For intracellular Dlg1 and WASp analysis, T cells were fixed and permeabilized with the Foxp3 Fixation/Permeabilization buffer set (eBioscience 00-5523-00), washed and stained for 30 minutes at 4°C with primary antibodies (Dlg1 or WASp at 1:1000). Cells were washed, stained for 30 minutes at 4°C with Alexa Fluor647 AffiniPure F(ab)2 Donkey Anti-Mouse IgG (1:5000). Cells were then washed, fixed in 2% paraformaldehyde and analyzed with a BD FACS-Caliber.

### Intracellular Cytokines

CTLs were restimulated with platebound anti-CD3 (2ug/mL) and anti-CD28 (2ug/mL) antibodies, EG.7 cells, or EL-4 cells pulsed with OVA_257-264_ peptide for 4hrs in the presence of BD GolgiPLUG. Cells were surface stained with CD8b-PE in FACS wash buffer (PBS + 3% FCS + 0.1% sodium azide) prior to overnight fixation and permeabilization with BD Cytofix/Cytoperm at 4°C. Cells were then washed in BD Perm wash solution, stained for 30 mins with IFNγ-APC or IL-2-APC, washed and data collected using a BD FACS-Calibur.

### Intracellular phospho-p38

For intracellular phospho-p38 analysis, cells were stimulated with plate-bound anti-CD3 (5μg/mL) and anti-CD28 (20μg/mL). After the stimulation, cells were immediately fixed in 4% PFA for 15mins on ice. Cells were then harvested, washed with FACS buffer and permeabilized with ice cold methanol overnight at 4°C. Cells were stained with Alexa Fluor647 Mouse anti-p38 MAPK (pT180/pY182) for 30mins at room temperature, washed and analyzed on a BD FACS-Calibur.

### Degranulation

Primary mouse OT-1 CTLs or OT-1 hybridoma T cells and EL-4 cells pulsed with OVA_257-264_ or EG.7 target cells were co-cultured (1:1) in 200μL media in 96-well U-bottom plates at 37°C for 3–4 hrs, in the presence of 1.0μL CD107a-APC, 1.0μL GolgiPLUG and 1.0μL GolgiSTOP. Cells were harvested, surface stained with CD8b-PE in FACS buffer, washed, fixed in 2% PFA and then analyzed using a BD FACS-Calibur

### Actin Polymerization

OT-1 CTLs were spun onto adherent MEF.B7.OVA cells in 12-well plates and placed in an incubator for 15mins. Cells were placed on ice and ice cold PBS was added. Cells were harvested, spun down and resuspended in 200μL of BD Cytofix/Cytoperm for overnight fixation and permeabilization at 4°C. Cells were then washed in BD Perm wash solution, stained for 1hr with Alexa Fluor 647 phalloidin (0.3 units) and CD8b-PE (1:200) in 200μL BD Perm wash solution, washed and fixed in 2% PFA. Data was collected using a BD FACS-Calibur.

### Conformational Opening of WASp

OT-1 hybridomas were stimulated with EG.7 cells (1:1) for 30mins and then immediately fixed in 4% PFA on ice for 15mins. Cells were then washed and surface stained with CD8b-PE. Cells were then permeabilized with BD Cytofix/Cytoperm, washed and stained for 45mins at 4°C with anti-WASp 26E6 (1:200). Cells were washed, stained for 30mins at 4°C with Alexa Fluor647 AffiniPure F(ab)2 Donkey Anti-Mouse IgG (1:1000). Cells were then washed and analyzed with a BD FACS-Caliber.

### Statistical Analysis

Standard deviations (SD) and standard error of the mean (SEM) were calculated using Excel (Microsoft). Statistical significance was determined by performing a two-tailed t test. p values < 0.05 were considered significant.

## Results

### T cells express two distinct Dlg1 protein variants due to alternative splicing

**Fig 1:**
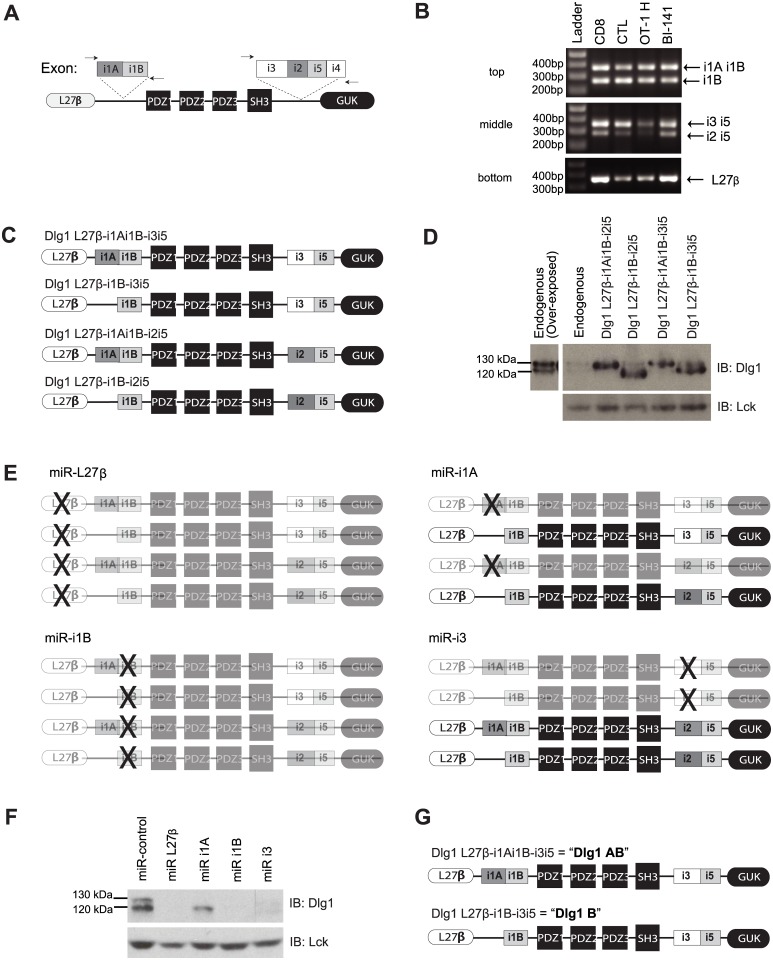
T lymphocytes express two major Dlg1 protein variants due to alternative splicing: Dlg1AB and Dlg1B. (A) Schematic of the analyzed areas of splicing in *dlg1*. (B) RT-PCR of cDNA from different murine T cells (CD8 = purified unstimulated primary OT-1 CD8+ T cells; CTL = purified OT-1 CD8+ T cell stimulated *ex vivo* with CD3/CD28 and expanded in rIL-2; OT-1H = CD8+ OT-1 hybridoma T cell line; BI-141 T hybridoma cell) using primers that flank the i1A/i1B (top) or i2/i3/i4/i5 (middle) splice region. Primers that lie within L27β were also used (bottom). (C) Schematic of four possible *dlg1* transcripts expressed in T cells. (D) BI-141 T cells infected with viruses encoding different Dlg1 splice variants were analyzed via western blotting for Dlg1 expression; Lck was used as a loading control. (E) Schematic representation of Dlg1 variants targeted for knockdown using miR-based knockdown viruses. (F) BI-141 T cells infected with knockdown viruses targeting L27β, i1A, i1B or i3 were analyzed via western blotting for Dlg1; Lck was used as a loading control. (G) Schematic representation of the two major Dlg1 protein variants expressed in T cells.

To investigate if alternative splicing of *dlg1* occurs in T cells we developed PCR primers that flanked the i1A/i1B or i3/i2/i5/i4 splicing regions (Fig 1A) and amplified cDNA from: freshly isolated CD8+ T cells, *in vitro* differentiated CTL, Th1, Th2 and T cell hybridomas, and DNA sequenced the PCR products. Assessing the i1A/i1B region we observed two RT-PCR products in all T cells surveyed, which based on size and DNA sequencing were found to be the i1B and i1Ai1B exon combinations (Fig 1B, top). All T cells surveyed also expressed the i3i5 and i2i5 splice combinations (Fig 1B, middle). Finally, all T cells expressed L27β (Fig 1B, bottom); CXCα was not assessed in this study. Similar results were also seen in other hematopoietic and non-hematopoietic cells (S1 Fig). Collectively, these findings identify at least four possible *dlg1* transcripts expressed in T cells, differing in their inclusion of i1A and the inclusion of i3 or i2 (Fig 1C).

To begin to explore which *dlg1* variants were expressed on the protein level, each of the four variant sequences were overexpressed in T cells. The migration distances of Dlg1 in resulting cells were compared to endogenous Dlg1, which runs as a protein doublet at 130kDa and 120kDa. The 130kDa band best corresponded with Dlg1 L27β-i1Ai1B-i3i5 or Dlg1 L27β-i1Ai1B-i2i5, and the 120kDa band best corresponded with Dlg1 L27β-i1B-i3i5 (Fig 1D).

To determine which of these Dlg1 variants were endogenously expressed, we utilized the previously described microRNA-based knockdown vector MGP [21]. We cloned four sequences into MGP that selectively targeted the L27β, i1A, i1B or i3 exons of Dlg1 and labeled these knockdown constructs: miR-L27β, miR-i1A, miR-i1B and miR-i3. We were unable to target the i2 exon as it was only 36bp. If all four Dlg1 variants were expressed in T cells miR-L27β would target all four, miR-i1A would target two (Dlg1 L27β-i1Ai1B-i3i5 and Dlg1 L27β-i1Ai1B-i2i5), miR-i1B would target all four, and miR-i3 would target two (Dlg1 L27β-i1Ai1B-i3i5 and Dlg1 L27β-i1B-i3i5) (Fig 1E). Targeting the L27β and i1B exons significantly decreased the expression of both Dlg1 protein bands, indicating that the majority of Dlg1 variants expressed in T cells indeed contained these exons. Targeting the i3 exon also decreased the expression of both Dlg1 protein bands, indicating that the majority of Dlg1 variants expressed the i3i5 exon combination, but not the i2i5 exon combination. However, when i1A was targeted a selective loss of the 130kDa Dlg1 protein variant was observed, indicating that the 130kDa Dlg1 variant contained i1A, while the 120kDa Dlg1 variant did not (Fig 1F). Similar results were observed in 3T3 fibroblasts (S1 Fig). Therefore, at least two major protein variants of Dlg1, which migrate as distinct bands on SDS PAGE, are expressed in T cells due to alternative splicing: Dlg1-L27β-i1Ai1B-i3i5 (Dlg1AB) and Dlg1-L27β-i1B-i3i5 (Dlg1B) (Fig 1G).

### Dlg1AB selectively associates with Lck and couples the TCR to optimal alternative p38 activation

**Fig 2:**
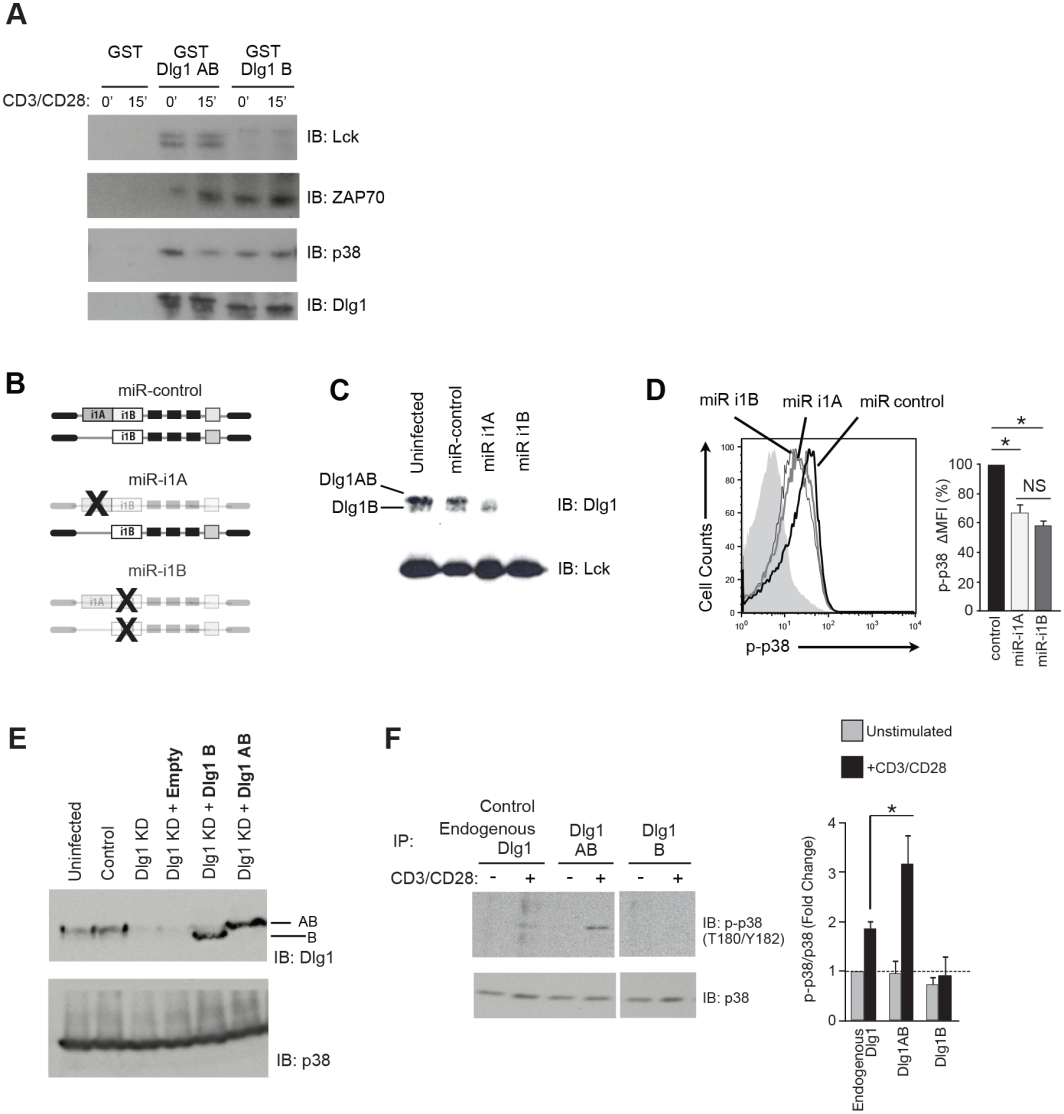
Dlg1AB selectively associates with Lck and supports optimal p38 phosphorylation while Dlg1B does not. (A) GST-tagged Dlg1AB and Dlg1B or GST-alone were incubated with protein lysate from unstimulated (0min) or stimulated (15min) T cells. Associated Lck, ZAP70 and p38 were identified by immunoblot. (B) Schematic representation of Dlg1 variants targeted for knockdown using miR-based knockdown viruses. (C-D) Purified primary OT-1 CD8+ T cells were stimulated with anti-CD3/anti-CD28 followed by infection with miR-based viruses. (C) SDS-PAGE of cell lysates assessing Dlg1 knockdown. (D) Cells were restimulated with anti-CD3/anti-CD28 for 30mins and stained for p-p38 (T180/Y182). CD8+GFP+ cells were gated and histograms of phosphorylated p38 are shown. The percentage of p38 phosphorylation relative to miR-control is quantified as ΔMFI (%), where ΔMFI = stimulated MFI—unstimulated MFI, and where miR-control is set to 100%. Error bars represent SD of means from three independent experiments. *p < 0.05. (E-F) OT-1 hybridoma T cells were infected with the indicated Dlg1 re-expression (bold) and/or Dlg1knockdown (KD) viruses. The Dlg1 knockdown (Dlg1 KD) construct targets the 3’UTR of *dlg1* allowing re-expression of specific Dlg1 splice variants. (E) Cells were analyzed via protein immunoblotting for Dlg1; p38 was used as a loading control. (F) OT-1 hybridoma T cells expressing endogenous Dlg1 or selectively re-expressing Dlg1AB or Dlg1B were left unstimulated (-) or stimulated with anti-CD3/anti-CD28 (+) for 15 mins, followed by immunoprecipitation with anti-Dlg1 and assessed for bound p38 T180/Y182 phosphorylation via immunoblotting (left). Fold change in densitometry of phospho-p38 relative to total p38, with the unstimulated endogenous Dlg1 condition normalized to 1.0 (denoted by the horizontal doted line) is shown (right). Error bars represent SD of means from three independent experiments. *p < 0.05.

Dlg1AB differs from Dlg1B by the presence of the proline-rich i1A domain. The i1A domain is predicted to bind the SH3-domain of Lck, which we previously demonstrated to be necessary for Dlg1association [5, 17, 25]. To determine if the i1A-domain was indeed required for Lck association we performed pulldown assays with GST-tagged Dlg1AB or Dlg1B. We found that only Dlg1AB could pulldown Lck, while both proteins could pulldown the PDZ-associated protein p38 and tyrosine kinase ZAP70 (Fig 2A).

Lck is required for the activation of the alternative p38 pathway. Specifically, Lck activates ZAP70 allowing for direct ZAP70 phosphorylation of p38 and downstream NFAT-dependent transcription [8, 9]. Thus, we hypothesized that Dlg1 splice variants may differentially regulate the alternative p38 pathway. To test this hypothesis, we utilized our microRNA-based knockdown system to diminish Dlg1AB expression (using miR i1A) or total Dlg1 expression (using miR i1B) in CD8+ CTLs (Fig 2B and 2C). We found a similar decrease in TCR-triggered phospho-p38 in cells lacking only Dlg1AB (miR i1A), and in cells lacking both Dlg1AB and Dlg1B (miR i1B); identifying Dlg1AB as a key regulator of optimal TCR-induced alternative p38 activation (Fig 2D). Additionally, we generated stable *dlg1* knockdown T cells by targeting the 3′UTR of *dlg1* and selectively re-expressed Dlg1AB or Dlg1B (Fig 2E). When Dlg1 complexes were isolated from these cells, we found both variants were able to associate with p38; however, only Dlg1AB facilitated optimal TCR-induced p38 phosphorylation (Fig 2F). Collectively, our results show that Dlg1AB orchestrates optimal alternative p38 activation through association with Lck.

### Dlg1AB promotes p38-dependent transcription of proinflammatory cytokines

**Fig 3:**
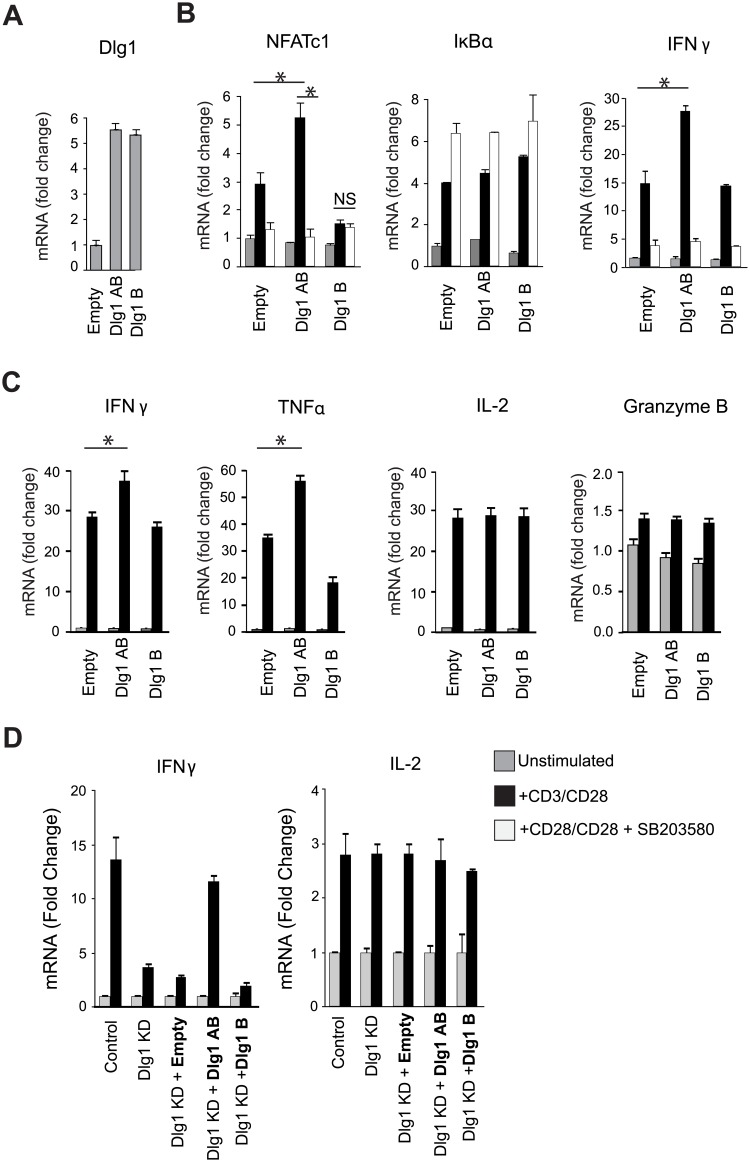
Dlg1AB selectively enhances NFAT-dependent transcription of IFNγ and TNFα but not IL-2 or granzyme B. (A-C) Purified primary OT-1 CD8+ T cells were stimulated with anti-CD3/anti-CD28 followed by infection with control (Empty) or Dlg1-viruses. (A) Cells were isolated and assessed for Dlg1 overexpression via qPCR. (B-C) Cells were restimulated with anti-CD3/anti-CD28 or left unstimulated in the presence or absence of 10μM p38 inhibitor (SB203580). RNA was isolated for qPCR analysis. mRNA was normalized to L32 and fold-increase in mRNA expression vs. unstimulated samples is shown. Error bars represent SD of samples analyzed in triplicate. *p < 0.05. Data are representative of three independent experiments. (D) CD8+ OT-1 hybridoma T cells infected with Dlg1 re-expression (bold) and/or Dlg1 knockdown (KD) constructs where stimulated with anti-CD3/anti-CD28 or left unstimulated. RNA was isolated for qPCR analysis. mRNA was normalized to L32 and fold-increase in mRNA expression vs. unstimulated samples is shown. Error bars represent SD of samples analyzed in triplicate.

TCR-triggered alternative p38 activation leads to NFAT-dependent transcription [8, 13]. In addition, the alternative p38 pathway has recently been demonstrated to regulate T cell-mediated autoimmunity and inflammation in vivo [26, 27]. Thus we tested the hypothesis that Dlg1 splice variants may have differential impacts in coordinating p38/NFAT-dependent gene expression of proinflammatory cytokines in CD8+ CTLs. First, we found that overexpression of Dlg1AB (Dlg1-L27β-i1Ai1B-i3i5), but not Dlg1B (Dlg1-L27β-i1B-i3i5), enhanced TCR-induced expression of the NFAT-dependent gene NFATc1, but not of the NFκB-dependent gene IκBα. Enhanced NFATc1 gene expression was prevented by pharmacological inhibition of p38 (Fig 3A and 3B). Furthermore, overexpression of Dlg1 L27β-i1Ai1B-i2i5 also enhanced TCR-induced expression of NFATc1, but not IκBα, indicating that substitution of the i3i5 exon combination for the i2i5 exon combination within the HOOK domain of Dlg1AB had no effect on NFAT-dependent gene expression (Fig 1C and S2 Fig). Overexpression of Dlg1AB, but not Dlg1B, also enhanced TCR-induced proinflammatory genes IFNγ and TNFα, but not IL-2 or granzyme B (Fig 3C). Pharmacologic inhibition of p38 activity blocked Dlg1AB-enhanced proinflammatory cytokine gene expression (Fig 3B). Therefore, we found that not all TCR-induced genes are equally impacted by Dlg1, and showed that Dlg1AB, but not Dlg1B, links the TCR to a subset of downstream signals (Lck/p38) and functions (IFNγ and TNFα, but not IL-2 or granzyme B gene expression).

**Fig 4:**
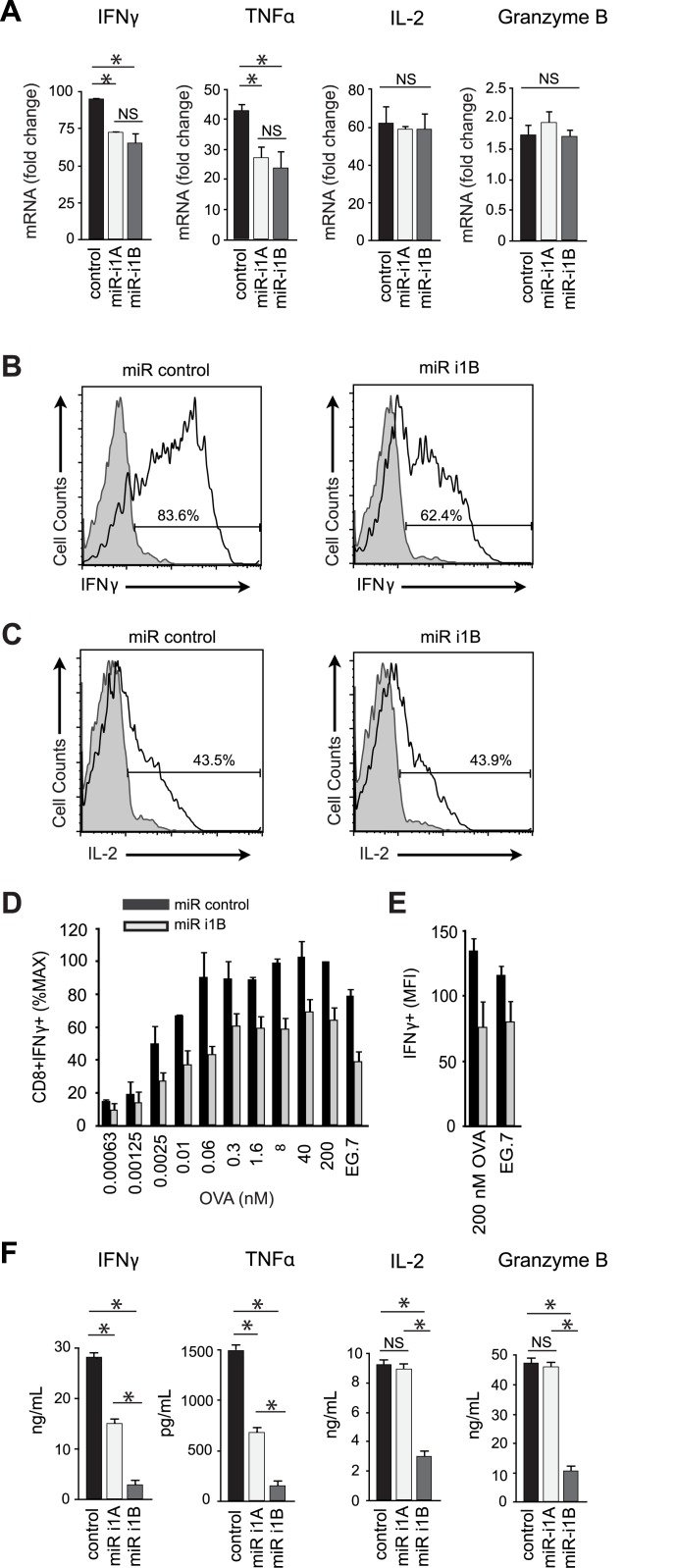
Dlg1AB knockdown diminishes transcription of IFNγ and TNFα, but not IL-2 or granzyme B, while total Dlg1 knockdown additionally impairs cytokine and granzyme B secretion. (A-F) Purified primary OT-1 CD8+ T cells were stimulated with anti-CD3/anti-CD28, followed by infection with miR-based viruses. (A) Cells were restimulated with anti-CD3/anti-CD28 for 4hrs or left unstimulated. RNA was isolated for qPCR analysis. mRNA was normalized to L32 and the fold-increase in mRNA expression vs. unstimulated samples is shown. Error bars represent SD of samples analyzed in triplicate. *p < 0.05. Data are representative of three independent experiments. (B-C) Cells were restimulated with anti-CD3/anti-CD28 for 4hrs (black line) or left unstimulated (filled histogram) in the presence of GolgiPLUG and assessed for intracellular cytokines. (D-E) Cells were restimulated with EG.7 cells or EL-4 cells pulsed with various concentrations of OVA_257-264_ peptide for 4 hrs in the presence of GolgiPLUG and assessed for intracellular cytokines. (D) CD8+IFNγ+ cells (%MAX) were measured by setting the miR-control 200nM OVA condition to 100%. The mean and SD of three independent experiments is shown. (E) The mean and SD for the MFI of IFNγ+ cells from three independent experiments is also shown.(F) Cells were restimulated with anti-CD3/anti-CD28 for 48hrs and supernatants collected for ELISA analysis. Error bars represent SD of samples analyzed in triplicate. *p < 0.05. Data are representative of three independent experiments.

We next examined whether Dlg1 knockdown impaired functionality in CD8+ CTLs. Loss of Dlg1AB reduced TCR-triggered IFNγ and TNFα gene expression to levels equivalent to total Dlg1 knockdown, while not affecting TCR-triggered IL-2 or granzyme B gene expression (Fig 4A). Accordingly, intracellular cytokine analysis demonstrated that total Dlg1 knockdown prevented optimal IFNγ protein production, but did not affect intracellular IL-2 protein production in response to anti-CD3/anti-CD28 or antigen at several antigen concentrations (Fig 4B–4E). We next examined TCR-induced IFNγ and IL-2 gene expression in T cells where endogenous Dlg1 was knocked down and selectively replaced with Dlg1AB or Dlg1B. Expression of Dlg1AB (but not Dlg1B) facilitated TCR-triggered IFNγ gene expression, while Dlg1 knockdown and/or re-expression had no affect on TCR-induced IL-2 gene expression (Fig 3D). Together, these results support a model in which Dlg1AB, but not Dlg1B, associates with Lck, promotes p38 phosphorylation and leads to p38-dependent proinflammatory cytokine gene expression in CD8+ T cells.

### Dlg1AB and Dlg1B support TCR-triggered p38-independent, actin-dependent degranulation

We previously reported that Dlg1 knockdown impairs CTL cytotoxicity and IL-2 secretion in CD8+ T cells [5]. In light of our findings that Dlg1 does not control granzyme B or IL-2 gene expression, we explored the possibility that Dlg1 controls the release of effector molecules such as granzyme B and IL-2 [28, 29]. Utilizing selective knockdown to assess the role of Dlg1 variants in effector release, we found that Dlg1AB knockdown decreased IFNγ and TNFα, but not IL-2 or granzyme B secretion. Additionally, knockdown of both Dlg1AB and Dlg1B further diminished IFNγ and TNFα secretion compared to Dlg1AB knockdown alone, and significantly decreased IL-2 and granzyme B secretion (Fig 4F). These findings suggest that Dlg1 coordinates two discrete TCR-triggered pathways that specify TCR-triggered effector gene expression and release. While Dlg1AB is required for regulation of proinflammatory cytokine gene expression, both Dlg1 variants coordinate TCR-triggered secretion of cytokines and lytic factors.

**Fig 5:**
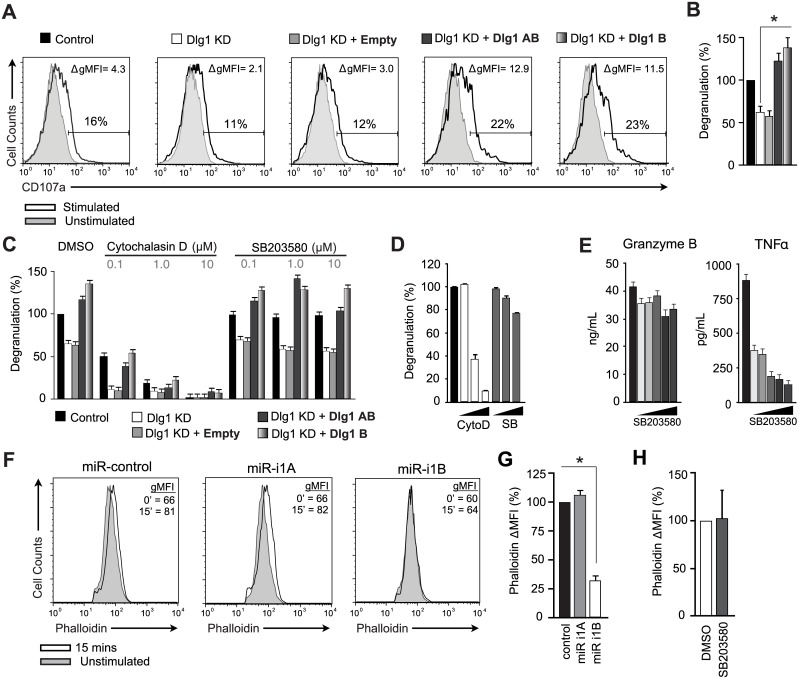
Dlg1 regulates TCR-triggered, p38-independent degranulation and actin polymerization. (A-C) OT-1 hybridoma CD8+ T cells infected with re-expression and/or knockdown constructs were stimulated with EG.7 cells in the presence of anti-CD107a. (A) Representative unstimulated (filled) and stimulated (line) histograms for each condition are shown. The percentage of CD107+ cells in each stimulated condition and the change in gMFI (stimulated gMFI- unstimulated gMFI) of the entire population are shown within each panel. (B) The percentage of degranulation relative to control is quantified as Degranulation(%), where Degranulation (%) = stimulated CD107+(%)–unstimulated CD107+(%), and where the control condition is set to 100%. Error bars represent SD of means from six independent experiments. (C-D) OT-1 hybridomas (C) or primary OT-1 CD8+ T cells (D) pretreated with DMSO, cytochalasin D (0.1, 1.0, 10 μM) or SB203580 (0.1, 1.0, 10 μM) were stimulated with EG.7 cells. Degranulation was quantified as Degranulation (%). Error bars represent SD of samples analyzed in triplicate. (E) Primary OT-1 CD8+ CTLs pretreated with DMSO or SB203580 (0.625, 1.25, 2.5, 5, 10 μM) were stimulated with anti-CD3/anti-CD28 for 48hrs. Supernatants were analyzed via ELISA. Error bars represent SD of samples analyzed in triplicate. (F-H) Primary OT-1 CD8+ T cells infected with miR-based viruses (F-G) or pretreated with 10μM SB203580 or DMSO for 30mins (H) were stimulated with MEF.B7.OVA cells for 15mins. Actin polymerization was assessed by phalloidin staining. (F) Histograms of phalloidin for CD8+GFP+ cells. (G-H) Change in actin polymerization was quantified as, ΔMFI = stimulated MFI—unstimulated MFI, and miR-control is set to 100%. Error bars represent SD of means from four independent experiments. *p < 0.05.

In CD8+ CTLs, actin-dependent release of granzyme B-containing lytic granules mediates contact-dependent cytotoxicity [29]. To explore a potential role for Dlg1 and p38 in regulating CTL degranulation, we examined the consequences of Dlg1 splice variant expression and pharmacologic inhibition in T cells where endogenous Dlg1 had been knocked-down and replaced with Dlg1AB or Dlg1B (Fig 5). We found that re-expression of Dlg1AB or Dlg1B rescued and enhanced antigen-induced degranulation, measured by exposure of CD107a on the T cell surface (Fig 5A and 5B). Pretreatment with varying concentrations of p38 inhibitor SB203580 had no effect on degranulation (Fig 5C). Similarly in primary OT-1 CTLs, p38 inhibition did not greatly affect degranulation or granzyme B secretion (Fig 5D and 5E). In contrast, pretreatment with actin inhibitor cytochalasin D prevented degranulation, indicating that polymerization of actin, but not p38, was required for degranulation (Fig 5C and 5D).

We next examined the effects of total versus select Dlg1AB knockdown on TCR-induced actin polymerization in CD8+ CTLs. While total Dlg1 knockdown impaired TCR-triggered actin polymerization, selective Dlg1AB knockdown had no effect; suggesting that actin polymerization like degranulation was p38-independent (Fig 5F and 5G). Accordingly, pretreatment of CTLs with p38 inhibitor SB203580 had no effect on antigen-induced actin polymerization (Fig 5H). Taken together these findings elucidate a p38-independent, actin-dependent pathway whereby both Dlg1 variants can promote CTL granule release.

### Dlg1 facilitates WASp activation to promote degranulation

**Fig 6:**
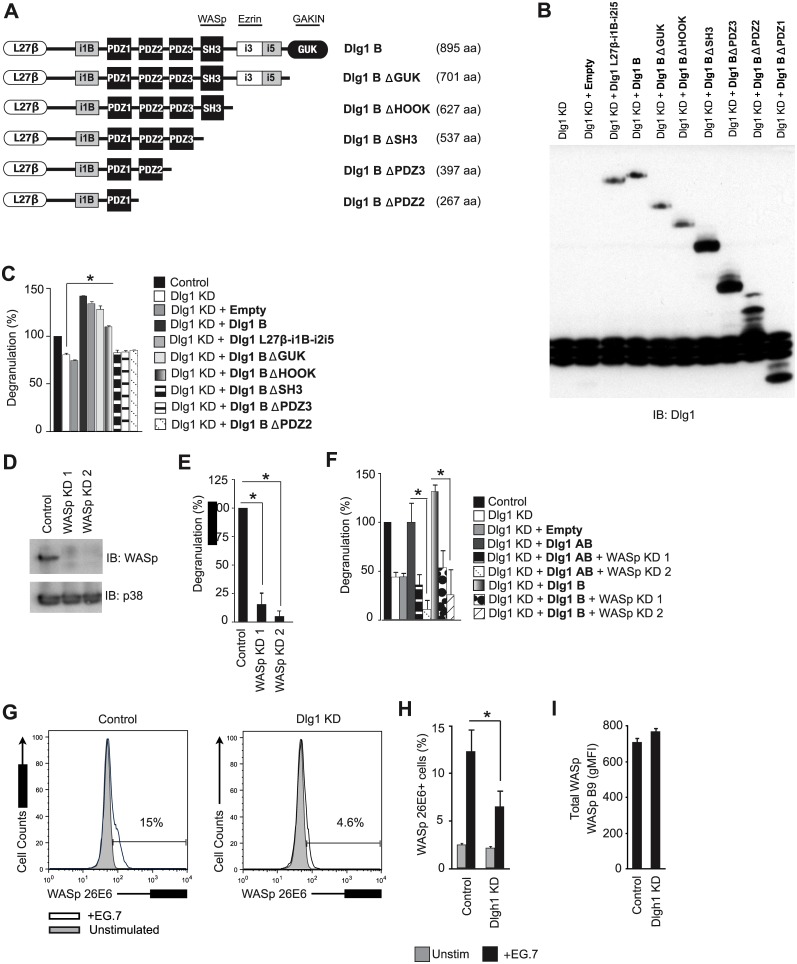
Dlg1-SH3 controls WASp activation to mediate degranulation. (A) Schematic representation of Dlg1B and Dlg1 B truncations, and possible interaction sites between cytoskeletal ligands. (B-I) OT-1 hybridoma CD8+ T cells were infected with re-expression (bold) and/or knockdown (KD) constructs. (B) Cells were analyzed via SDS-PAGE and immunoblotted for Dlg1. (C,E,F) Cells were stimulated with EG.7 cells in the presence of anti-CD107a. The percentage of degranulation relative to control is quantified as Degranulation(%), where Degranulation (%) = stimulated CD107+(%)–unstimulated CD107+(%), and where the control condition is set to 100%. Error bars represent SD of means from three independent experiments; *p < 0.05. (D) Protein lysates from control and WASp knockdown cells were analyzed via SDS-PAGE and immunoblotting to assess WASp knockdown; p38 was used as a loading control. (G-H) Cells were left unstimulated (grey) or stimulated with EG.7 cells for 30 mins (black) and intracellularly stained with anti-WASp 26E6 (which recognizes “open” WASp). (G) Representative histograms are shown. (H) Average percentage of cells with “open” WASp with error bars representing SD of means from three independent experiments. *p < 0.05. **(I)** Cells were stimulated with EG.7 cells for 30 mins and intracellularly stained for total WASp protein levels. Error bars represent SD of samples analyzed in triplicate. Data are representative of at least three independent experiments.

Since several cytoskeletal effectors that may contribute to degranulation associate with the C-terminus of Dlg1, we generated T cells that expressed Dlg1B (Dlg1 L27β-i1B-i3i5) or Dlg1B C-terminal truncations, and assessed their ability to promote degranulation (Fig 6A–6C) [8, 30, 31]. Re-expression of full length Dlg1B or Dlg1B proteins lacking the GUK and/or HOOK domains (Dlg1B ΔGUK, Dlg1B ΔHOOK) rescued and enhanced degranulation levels above those observed in cells expressing endogenous Dlg1. Re-expression of Dlg1 L27β-i1B-i2i5 also recused and enhanced degranulation, indicating that substitution of the i3i5 exon combination for the i2i5 exon combination within the HOOK domain of Dlg1B had no effect on Dlg1-mediated degranulation. Conversely, expression of truncated Dlg1B proteins lacking the SH3 domain (Dlg1B ΔSH3, Dlg1B ΔPDZ3, Dlg1B ΔPDZ2) were unable to rescue the effects of Dlg1 knockdown on degranulation despite being expressed at similar levels (Fig 6A–6C and S3 Fig).

We have demonstrated that WASp, a known mediator of F-actin polymerization, can directly bind a Dlg1SH3 fragment. Therefore we explored WASp as a potential Dlg1SH3 effector of actin-dependent degranulation [8, 32]. Knockdown of WASp using two target sequences dramatically reduced antigen-induced degranulation in T cells (Fig 6D and 6E). Furthermore, re-expression of Dlg1 variants in Dlg1/WASp double knockdown cells was unable to rescue antigen-induced degranulation, suggesting that WASp plays a role in Dlg1-mediated degranulation (Fig 6F and S3 Fig).

WASp is held in an autoinhibited closed state by intra- or intermolecular interactions between the VCA and GBD domains. Disruption of the VCA-GBD interaction results in opening and activation of WASp. We hypothesized that Dlg1 may open WASp by binding its proline-rich domain, like other SH3-containing proteins [33]. To test this hypothesis we utilized a conformation-specific antibody which recognizes the open conformation of WASp [34]. Total Dlg1 knockdown led to a decrease in the number of cells with open WASp after TCR stimulation with antigen, while not affecting total WASp protein levels (Fig 6G–6I). Together these data identify Dlg1 as a novel regulator of WASp activation, and suggest that Dlg1SH3 association with WASp may contribute to p38-independent degranulation.

## Discussion

CD8+ T cells show a broad range of functionality, with differing capacities to produce and secrete a spectrum of effector molecules in response to TCR triggering [3]. Pathogens evoke specific constellations of functionally distinct T cells, a phenomenon proposed to optimize T cell responsiveness for eradicating a given pathogen. Acquisition and maintenance of CD8+ poly-functionality is correlated with an increased quality of T cell response to *Leishmania*, adenovirus, vaccinia virus, HIV and tumors [35–38]. Alternatively, in response to influenza, the development of lung infiltrating CTLs lacking proinflammatory cytokine responsiveness is correlated with better clinical course [1, 39]. Still, in other instances, development of CD8+ T cells with partial functionality enables persistent infection though induction of T cell exhaustion [40]. Despite our growing knowledge of CD8+ subpopulations and their impact on health and disease, little is known about how CD8+ T cells customize TCR-triggered signaling to produce functional diversity. Here we demonstrated that Dlg1 splice variants couple TCR engagement to proinflammatory cytokine production and/or degranulation, elucidating one possible mechanism of diversifying T cell functional outcome within the CD8+ T cell population.

We and others have demonstrated that Dlg1 plays a vital role in T cell development, activation and effector responses utilizing knockdown and overexpression methods *in vitro* [5, 8, 11, 13, 31, 41]. However, initial Dlg1 knockout studies have been unremarkable, possibly due to compensation of other Dlg family members or scaffolds during development [11, 41, 42]. Supporting this hypothesis, and validating our present and previous Dlg1 knockdown studies, we have recently demonstrated that acute *in vitro dlg1* knockout in CD8+ T cells containing loxP sites flanking the *dlg1* exon encoding a portion of PDZ1 and PDZ2 treated with Cre-recombinase were defective in p38 phosphorylation, induction of proinflammatory cytokine gene expression and CTL cytotoxicity [43]. Future studies utilizing acute Dlg1 knockout mice should allow for a more accurate assessment of the functional role of Dlg1 in T lymphocytes and other hematopoietic cell populations.

In this study, we identified two discrete TCR signaling pathways regulated by Dlg1 in CD8+ T cells: Dlg1:p38-dependent production of proinflammatory cytokine gene expression and Dlg1:p38-independent degranulation. We also discovered that T cells express at least two major protein variants of Dlg1 due to alternative splicing: Dlg1AB (Dlg1-L27β-i1Ai1B-i3i5) and Dlg1B (Dlg1-L27β-i1B-i3i5). While we did observe i2-containing *dlg1* transcripts by RT-PCR analysis, targeting the i3-exon for knockdown significantly reduced Dlg1 protein levels, suggesting that if i2-containing Dlg1 protein variants are indeed expressed in T cells they constitute a small fraction of the total pool of Dlg1 proteins (Fig 1). Furthermore, this study demonstrated that substituting the i2-exon for the i3-exon does not affect TCR-triggered Dlg1-mediated NFATc1 gene expression or degranulation, suggesting that at least in the functions examined in this study there seems to be no functional difference between i2-containing and i3-containing Dlg1 protein variants (S2 Fig and Fig 6).

We found that Dlg1AB, but not Dlg1B, bound Lck and coupled TCR engagement to optimal p38 phosphorylation (Fig 2). We hypothesize that Dlg1B can not support optimal p38 activation because Dlg1B-bound ZAP70 is in an auto-inhibited or inactive/non-fully active state, as a result of the lack of Lck binding, lack of proper protein kinase positioning and/or lack of direct ZAP70 phosphorylation by Lck. This hypothesize is supported by previous work demonstrating that lack of Lck protein is sufficient to abrogate alternative p38 phosphorylation *in vivo* [9]. Another possibility is that Dlg1B-bound ZAP70 is activated by a non-Lck-dependent mechanism but is mal-positioned relative to p38, preventing optimal p38 phosphorylation. This lack in proper tyrosine kinase positioning may be caused by the spatial-dimensional absence of the i1A domain in Dlg1B, or the lack of Lck-dependent phosphorylation of Dlg1B on Y222, which has been proposed to serve as a conformational switch to open the Dlg1 scaffold to allow for the binding of additional ligands or proper juxtaposition of already bound ligands [43]. These findings do not exclude the possibility that Dlg1B allows for suboptimal and/or delayed p38 phosphorylation that is independent of Dlg1-associated Lck. Fyn has been demonstrated to directly phosphorylate p38 *in vitro*, and has a delayed activation kinetic in T cells relative to Lck [44, 45]. However, because Fyn does not associate with Dlg1, we favor a model in which Lck binding to Dlg1AB is of primary importance for Dlg1-medicated p38 activation [5, 25]. Consistent with this suggestion, Dlg1AB supports optimal p38-dependent production of downstream pro-inflammatory cytokines. Future experiments exploring additional potential roles of Dlg1B and/or Fyn in altering p38 activation kinetics can help resolve these issues, though the data presented make clear that Dlg1AB is primarily responsible for alternative p38 activation previously attributed to Dlg1 scaffolding activity [8].

Whereas Dlg1AB uniquely associated with Lck and orchestrated optimal alternative p38 activation and proinflammatory cytokine production, both Dlg1AB and Dlg1B supported p38-independent granule release. Dlg1AB differs from Dlg1B by the presence of the proline-rich i1A domain containing ten proline residues predicted to form two polyproline helical domains, one within the i1A domain and one at the junction between i1A and i1B, that may facilitate direct interaction with SH3-containing proteins including Lck (18, 26). While we hypothesize that one or both polyproline helical domains contribute to direct interaction with LckSH3, we can not rule out the possibility that i1A allows for a specific conformational change or post-translational modification of Dlg1 to allow for direct Lck binding somewhere outside the i1A domain. Interestingly, we have recently demonstrated that the i1A domain allows for Dlg1 tyrosine phosphorylation, which in turn facilitates optimal alternative p38 activation and proinflammatory gene expression [43]. Future studies assessing the relative contribution of each polyproline residue to Lck binding will aid in the understanding of Dlg1 structure and regulation.

We found that Dlg1AB, but not Dlg1B, selectively coupled TCR engagement to NFAT, but not NFκB, dependent transcription. Further, we found that Dlg1AB coordinated transcriptional activation of proinflammatory cytokines IFNγ and TNFα, but not IL-2 or granzyme B. These findings support reports that granzyme B gene expression is regulated by p38/NFAT-independent NFκB activation and that proinflammatory cytokine IFNγ and TNFα, but not IL-2 gene expression is regulated by alternative p38 activation [46, 47]. While transcription factor NFATc2 (NFAT1) is known to regulate IFNγ, TNFα, and IL-2 gene expression, only IFNγ and TNFα expression were impacted by Dlg1 knockdown. Dlg1 regulates transcription factor NFATc2 (NFAT1), but not NFATc1 (NFAT2), through p38-dependent phosphorylation of a regulatory serine found in the transactivation domain of NFATc2 [8]. Previous work has shown that NFATc2 regulates both TNFα and IL-2 gene expression, while NFATc1 regulates only IL-2 gene expression [48]. Therefore, we predict that NFATc1 compensates for NFATc2 in Dlg1-deficient cells enabling IL-2 gene expression.

Polarity proteins and cytoskeletal regulators have been hypothesized to act cooperatively to control lytic factor degranulation and cytotoxicity in CD8+ T lymphocytes [49]. Dlg1 and WASp are both required for optimal contact-dependent cytotoxicity, and control key processes required for lytic factor degranulation including lytic synapse formation/reformation, actin polymerization, and MTOC dynamics [5, 28, 31, 50, 51]. Here, we discovered a possible molecular link between Dlg1 and WASp that promotes TCR-triggered degranulation. We previously established WASp as a direct Dlg1 ligand, and mapped the association to the SH3-HOOK domain of Dlg1 [8]. Here we demonstrate that Dlg1 controls WASp opening and activation, and that Dlg1-mediated degranulation depends on Dlg1 fragments containing an intact SH3 domain. We propose that Dlg1 is one SH3-containing protein that may associate with the proline-rich domain of WASp via either canonical (tryptophan-tryptophan) or non-canonical binding, to disrupt WASp VCA-GBD interactions; allowing WASp-dependent actin polymerization, granule polarization and release of lytic factors. Dlg1 may additionally oligomerize, cluster and/or localize WASp; aggregating lipid rafts and TCRs at the immunological synapse [52]. Additionally, findings that Dlg1 knockdown diminished IL-2 secretion without affecting IL-2 gene or protein expression point to a potential role for Dlg1:WASp regulation of TCR-triggered cytokine secretion, in keeping with published data elucidating a requirement for WASp in TCR-triggered polarized secretion of IL-2 and IFNγ [53]. Data demonstrating that levels of secreted IFNγ and TNFα are more significantly diminished by total Dlg1 versus Dlg1AB knockdown (despite equivalent effects on gene expression) supports this suggestion. In support of Dlg1SH3 being one of several proteins that can activate WASp to promote WASp-dependent degranulation and possibly WASp-dependent cytokine release, we found that WASp-knockdown T cells had a more severe degranulation defect compared to Dlg1-knockdown T cells. Future studies determining the precise canonical and/or non-canonical binding site(s) within the SH3 domain of Dlg1 for WASp will aid in a more complete understanding of Dlg1-mediated WASp-dependent degranulation, and possibly TCR-triggered polarized secretion of IL-2 and IFNγ.

We have reported that Dlg1 can also regulate CD4+ T cells; selectively promoting Th1 cytokines, while impairing Th2 cytokine production [11]. Here we demonstrate that *dlg1* i1Ai1B, i1B, and i2i5, i3i5 exon combinations are expressed in Th1 and Th2 cells (S1 Fig). While alternative p38 and WASp activation have similarly been implicated in Th1/Th2 skewing, which Dlg1 splice variants and Dlg1-guided processes are responsible for specifying TCR-triggered functionality in CD4+ cells remains unclear [11, 13, 26, 54]. We hypothesize that Dlg1 splice variants play a major role in diversifying and specifying T cell responsiveness. Interestingly, a recent report has demonstrated that the Dlg1 i1A exon is within the top 1–2% of exons excluded from mRNA transcripts in human T cells following activation [55], highlight the potential for regulation of total *dlg1* expression and alternative splicing as T cell intrinsic mechanisms for diversifying TCR triggered effector functions. Future studies aimed at understanding Dlg1 activity and regulation in various T cell effector and memory populations will test this hypothesis. Through understanding the detailed molecular mechanisms involved in diversifying TCR signaling and functionality, we aim to identify novel therapeutic pathways and aid in basic understanding of T cell regulation and function.

## Supporting Information

S1 Fig Dlg1 alternative splice variants expressed by hematopoietic and non-hematopoietic cells. (A) RT-PCR of cDNA from different murine hematopoietic and non-hematopoietic cells (CD4+ Th1 skewed cells, CD4+ Th2 skewed cells, A20 B cell line, WeHi B cell line; RAW267 macrophage cell line; 3T3 fibroblasts) using primers that flank the i1A/i1B (top) or i2/i3/i4/i5 (middle) splice region. Primers that lie within L27β were also used (bottom). (B) 3T3 fibroblasts infected with miR-based knockdown viruses targeting specific regions of Dlg1 and analyzed for Dlg1 protein expression via immunoblotting; p38 was used as a loading control.
(EPS)

S2 Fig The i3 domain of Dlg1 is not required for NFAT-dependent gene expression. (A-B) BI-141 hybridoma T cells were infected with control (Empty) or Dlg1-viruses. Cells were stimulated with anti-CD3/anti-CD28 or left unstimulated. RNA was isolated for qPCR analysis of NFATc1 (A) and IκBα (B). mRNA was normalized to L32 and fold-increase in mRNA expression vs. unstimulated samples is shown. Error bars represent SD of samples analyzed in triplicate. Data are representative of at least two independent experiments.
(EPS)

S3 Fig Dlg1B truncations and WASp knockdown in OT-1 hybridoma T cells. (A-C) OT-1 hybridoma T cells were infected with the indicated Dlg1 knockdown (KD) and/or re-expression (bold) viruses. The Dlg1 knockdown (Dlg1 KD) construct targets the 3′UTR of *dlg1* allowing for re-expression of specific Dlg1 variants. The indicated cells were analyzed for intracellular Dlg1 (A, C) or WASp (B) protein levels via flow cytometry; geometric mean fluorescent intensities (gMFIs) are shown.
(EPS)

S1 Table Cloning, RT-PCR and qPCR primers. (DOCX)
