# Supplementary material for: Discs Large Homolog 1 Splice Variants Regulate p38 –Dependent and –Independent Effector Functions in CD8+ T Cells
Source: PLoS One. 2015 Jul 17;10(7):e0133353. doi: 10.1371/journal.pone.0133353 (PMC4505885; doi:10.1371/journal.pone.0133353)
Supplement: S1 Table — (DOCX) [file pone.0133353.s004.docx]

S1 Table. Cloning, RT-PCR and qPCR primers

| **RT-PCR Primer** | **Sequence** |
| --- | --- |
| Forward i1A/i1B | CGGTATCAGGATGAAGAGGTA |
| Reverse i1A/i1B | CCCCTTTCAAGTGTGATTTCC |
| Forward i2/i3/i4/i5 | CAGGCAGGTCACCCCAGA |
| Reverse i2/i3/i4/i5 | GGTCCTAATATGATGACTGGTCGGG |
|  |  |
| **qPCR Primer** | **Sequence** |
| Forward NFATc1 | GCCTCGTATCAGTGGGCGAAG |
| Reverse NFATc1 | CGAAGCTCGTATGGACCA |
| Forward IκBα | CTGCAGGCCACCAACTACAA |
| Reverse IκBα | CAGCACCCAAAGTCACCAAGT |
| Forward IFNγ | GTCAACAACCCACAGGTCCAG |
| Reverse IFNγ | CCTTTTCCGCTTCCTGAGG |
| Forward TNFα | AATGGCCTCCCTCTCATCAGT |
| Reverse TNFα | GCTACAGGCTTGTCACTCGAATT |
| Forward IL-2 | CCTGAGCAGGATGGAGAATTACA |
| Reverse IL-2 | TCCAGAACATGCCGCAGAG |
| Forward Granzyme B | AAACGTGCTTCCTTTCGGG |
| Reverse Granzyme B | GAAACTATGCCTGCAGCCACT |
| Forward Dlgh1 | AGATCGCATCATATCGGTGAA |
| Reverse Dlgh1 | TCAAAACGACTGTACTCTTCGG |
| Forward L32 | AAGCGAAACTGGCGGAAAC |
| Reverse L32 | TAACCGATGTTGGGCATCAG |
|  |  |
| **Cloning Primers** | **Sequences** |
| Forward XhoI Dlgh1 | ATCGCAATTGCACGAGCATGCCGGTCCGGAAGGAAGATACC |
| Reverse EcoRI Dlgh1 | ATTCGTGAATTCACTTCATAGCTTTTCTTTCGCTGGGTCCCAGATGTA |
| Forward BstI Dlgh1 | GGGTCTCTTCGAACCAGCCAGAAG |
| Reverse BstI Dlgh1 | CTTCTGGCTGGTTCGAAGAGACCC |
| Forward EcoRI Dlgh1 | TATGAATTCCTGCCGGTCCGG |
| Reverse SalI Dlgh1 | ACTGTCGACACTTCATAGCTTTTCTTTCGCTGGGTC |
| Reverse EcoRI Dlgh1 ΔGUK | TCATTGGAATTCTTTTCATTCTTGTTGATTCACTGGCTCATA |
| Reverse EcoRI Dlgh1 ΔHOOK | TCATTGGAATTCTTTTCAGAATTTGACCGTTTTTAATCGGGC |
| Reverse EcoRI Dlgh1 ΔSH3 | TCATTGGAATTCTTTTCAACTGACACTGCTATTCATCATCTG |
| Reverse EcoRI Dlgh1 ΔPDZ3 | TCATTGGAATTTTCATCCAGCTTCTTTTAATGCCTCCAC |
| Reverse EcoRI Dlgh1 ΔPDZ2 | TCATTGGAATTCTTTTCATCCAGCTTCTTTTAATGCCTCCAC |
|  |  |
